# Psychometric Properties of the Patient Health Questionnaire-9 in Elderly Chilean Primary Care Users

**DOI:** 10.3389/fpsyt.2020.555011

**Published:** 2020-11-17

**Authors:** Joseph Aslan, Félix Cova, Sandra Saldivia, Claudio Bustos, Carolina Inostroza, Paulina Rincón, Camila Ortiz, Vasily Bühring

**Affiliations:** ^1^Doctoral Program in Psychology, Universidad de Concepción, Concepción, Chile; ^2^Department of Psychology, Faculty of Social Sciences, Universidad de Concepción, Concepción, Chile; ^3^Department of Psychiatry and Mental Health, Faculty of Medicine, Universidad de Concepción, Concepción, Chile; ^4^Master Program in Politics and Government, Universidad de Concepción, Concepción, Chile; ^5^Master Program in Psychology, Universidad de Concepción, Concepción, Chile

**Keywords:** depression, patient health questionnaire, primary health care, sensitivity, specificity

## Abstract

**Background:** This study aimed to assess the measurement properties (reliability, factor structure, and criterion validity) of the Patient Health Questionnaire (PHQ-9) as an instrument for screening major depressive disorder (MDD) in elderly primary care users in Chile.

**Method:** About 582 participants aged between 65 and 80 years were enrolled from primary care centers. They completed the Composite International Diagnostic Interview (CIDI), a survey with sociodemographic characteristics and the PHQ-9.

**Results:** The PHQ-9 revealed an acceptable internal consistency (ω = 0.79 [95% CI: 0.75–0.80] and α = 0.78 [95% CI: 0.75–0.81]); confirmatory factor analysis demonstrated a good fit for both 1- and 2-factor solutions. The chi-square difference test (χ^2^ = 0.61, *gl* = 1, *p* = 0.43) and correlation between the somatic and the cognitive-effective latent factors were very high (*r* = 0.97, *p* < 0.001), indicating that the 1 factor model was more parsimonious. Utilizing the CIDI as the gold standard, the area under the curve (AUC) was 0.88 (SE = 0.04, 95% CI: 0.84–0.90). The optimal cut-off score of ≥ 6 yielded good sensitivity and specificity for detecting MDD (0.95 and 0.76, respectively). However, considering the clinical utility index, the cut-off score of ≥9 proved to be a more effective marker for discarding cases of MDD.

**Conclusion:** The PHQ-9 has adequate psychometric properties for elderly primary care users. In clinical settings, it showed its greatest utility in ruling out the presence of an MDD, however, its clinical value for identifying possible cases of MDD is limited. In cases above the cut-off point, it is recommended to perform a more thorough evaluation.

## Introduction

Late-life depression is one of the most frequent mental health disorders in older adults and is associated with poorer physical health, more limited social functioning, increased suicide risk, and higher overall mortality. It is also associated with other mental health problems and disorders such as anxiety disorders, substance and medication abuse as well as cognitive deficits (1). Unfortunately, it is frequently under-recognized, mainly because its clinical indicators, such as depression, sadness, tiredness, anhedonia, sleep disturbances, hypochondriasis, subjective cognitive complaints (e.g., poor concentration and memory) are confounded with normal expressions related to the aging process (2).

Preventing, identifying, and treating depression in a timely and adequate manner in older adults are relevant health objectives. Primary health care (PHC) plays an important role in the early detection and treatment of depressive disorders. Chilean national studies report a 24.4% annual prevalence for common mental disorders in PHC center users in the south-central area of the country, where major depressive disorder (MDD) was present in 13.4% of users (3). However, few studies estimate the prevalence of depression in older adults in PHC and, although this would be somewhat lower than in other age groups, it is still high (4).

Although clinical interviews are indispensable for proper diagnosis and evaluation of the symptoms associated with depressive disorders, simple and brief questionnaires are beneficial for screening and monitoring the trajectory of symptoms over time (5). Screening instruments need to demonstrate evidence of their reliability and validity in various contexts of use, without which their scores are unintelligible (6). The Patient Health Questionnaire (7) (PHQ-9) is one of the most popular depression measures internationally, in clinical and population based settings (8). Despite its brevity it has proven its ability to identify symptoms of depressive disorders, high screening capacity, and sensitivity to change in monitoring the treatment response (9, 10).

Various studies have analyzed the psychometric properties of the PHQ-9 in the elderly. There are studies in the United States (11, 12), China (13–15), Taiwan (16), United Kingdom (17, 18), Germany (19), Netherlands (20), Australia (21), and Brazil (22).

The PHQ-9 uses as an original recommendation—a cut-off score of 10—to detect a depressive disorder in the general population. Nevertheless, recent evidence regarding the optimal cut-off score for screening depression found that scores between 8 and 11 had satisfactory properties (8). In Chile, the PHQ-9 has been used in PHC contexts, showing evidence of good reliability and screening accuracy, with the original and lower cut-off scores (23, 24). Some authors argue that in the elderly, a lower cut-off score in the PHQ-9 would augment both sensitivity and specificity (20).

Concerning the structural validity of the PHQ-9, there is evidence corroborating the unidimensional structure of the scale (11, 24–30). However, some evidence has been found supporting a two-dimensional solution (16, 18, 19, 31). In these studies, one factor of the PHQ-9 represents non-somatic (cognitive and affective) depression symptoms, and another the somatic symptoms.

To our knowledge, the PHQ-9 screening characteristics have not yet been validated with the Chilean elderly in PHC. The objective of this study is to analyse the psychometric properties of the PHQ-9 in a Chilean elderly sample, in a PHC setting. Specifically, this investigation focuses on reliability among the PHQ-9 items (internal consistency), the dimensionality of the test (structural validity), and its convergence with a reference to a gold standard in identifying elderly patients with MDD (criterion validity).

## Methods

### Subjects and Procedure

This study adopted a descriptive, cross-sectional design.The study subject data were obtained to perform a sub analysis of the baseline sample of a larger study (32). The sample was recruited from July to August 2018, consisting of self-dependent men and women between the ages of 65 and 80, attending 15 PHC centers in two catchment areas in the central-southern part of Chile (Concepción and Talcahuano). The potential participants (*N* = 1,220) were contacted and invited during a home visit; 582 accepted (a 52.3% rejection rate), and an interview was coordinated for data collection. Those who agreed to participate provided written informed consent.

The inclusion criteria of our study were as follows: (1) aged between 65 and 80, (2) self-dependent and (3) user of a PHC center. Exclusion criteria were the following: (1) presence of a severe mental disorder (psychosis or bipolar disorder), (2) mental disability or dementia or (3) a disability preventing communication. The assessment of these criteria was made from the health records using a standardized instrument (EMPAM) (33).

Data collection was done over a 1-month period, through 40-min-long face-to-face interviews by trained research assistants at home visits. The survey included sociodemographic characteristics, the PHQ-9, the Composite International Diagnostic Interview (CIDI) and other measures not related to this study. The participants fulfilling the diagnostic criteria for MDD were referred to their PHC center.

## Measures

### Patient Health Questionnaire (PHQ-9)

The PHQ-9 is a self-questionnaire consisting of nine items that assess the presence and severity of depressive symptoms based on the DSM-IV criteria for MDD (34). It refers to symptoms experienced by patients during the 2 weeks preceding to the interview. In this study, the Spanish version of the scale was employed (35). The PHQ-9 scoring consists of a Likert scale: 0 (not at all), 1 (several days), 2 (more than half the days), and 3 (nearly every day). Total scores range from 0 to 27. The severity of symptoms can be organized into four categories: 0–4 (minimum), 5–9 (mild) 10–14 (moderate), 15–19 (moderate to severe), 20–27 (serious) (7). The PHQ-9 was developed as a screening tool, with recommended cut-off scores between 8 and 11 for a probable case of MDD (8).

### Composite International Diagnostic Interview (CIDI)

The CIDI (36) is a structured diagnostic interview developed by the World Health Organization (WHO). It has been used in epidemiological studies in the general population, with high inter-rater and test-retest reliability, and is a valid gold standard for MDD diagnosis (37, 38), with evidence of validity in multiple international studies, including Chile (4, 39, 40). The CIDI can be administered by lay interviewers, thus overcoming the limitations of interviews conducted only by professionals, maintaining the objective that the diagnoses strictly adhere to the established diagnostic criteria (41). CIDI 2.1 delivers diagnoses following the DSM-IV and ICD-10 criteria, for a life-long disorder, for last 12 months, and for the last 30 days (36, 42). For this study, the diagnoses were used according to DSM-IV criteria and their presence was evaluated during the last 6 months. Sections A (sociodemographic data) and E (depression) were used in this study.

### Data Analysis

For descriptive data and internal consistency analyses (measured by Cronbach's alpha and McDonald's omega coefficient) JASP software (43) was utilized. For criterion validity, we assessed the PHQ-9 performance in comparison to the gold standard using MedCalc 14.8 (44). The CIDI, which is used for the diagnosis of an MDD, was used as a criterion standard. The receiver operating characteristic curve (ROC) and area under the curve (AUC) were constructed against the presence of MDD by the CIDI. The following test characteristics of the PHQ-9 were compared with the CIDI: sensitivity, specificity, predictive values, likelihood ratios, clinical utility index, and the Youden J index. For the confirmatory factorial analysis (CFA), Mplus 8.4 software (45) was used. A unifactorial and bifactorial model were postulated, using the robust weighted least squares estimator (WLSMV), which does not assume the normality of the variables and is considered the best option to model categorical or ordinal data (46). A chi-square difference test was performed to determine which of the models has a best fit to the data using the DIFFTEST command.

## Results

### Participant Characteristics

We excluded from the study 5 of the 582 respondents due to incomplete data. The remaining 577 (99.14%) cases were included in the analyses. The mean age of the 577 participants was 71.77 years (SD = 4.13). Table 1 ilustrates the demographic characteristics of the sample. There were 374 women (64.8%) and 203 men (35.2%); 302 respondents were married (52.3%), 213 were bereaved or divorced (36.9%), and 62 (10.7%) were never married; 114 (19.9%) of the participants had worked in the last 12 months, 84 (14.6%) lived alone and 366 (63.7%) had <12 years of education.

**Table 1:** Socio-Demographic characteristics of the sample (*N* = 577).

		***N***	**%**
**Demographics**			
Gender	Men	203	35.0
	Women	374	65.0
Age Group	64–69	206	36.0
	70–79	349	61.0
	80+	22	4.0
Marital Status	Married	302	52.3
	Widow	138	23.9
	Divorced	75	13.0
	Never Married	62	10.7
Studies	Basic Education or incomplete secondary education	366	63.0
	Complete secondary education	104	18.0
	Higher Education	105	18.0
	Missing Data	2	0.3
Employment Situation	Full time work	32	5.5
	Part time work	66	11.4
	No work	479	83.0

The mean score of the 577 participants was 3.99 (SD = 4.31), with a range of 0 to 24. The median score was 3, with a skewness of 1.51 (SE = 0.10) and kurtosis of 2.20 (SE = 0.20). The distribution of the PHQ-9 scores is shown in Table 2. Most of the participants (87.9%) had a PHQ-9 score under the most common cut-off score (<10), and only 4.3% had a score of 15 or higher.

**Table 2:** Distribution of PHQ-9 scores and 6-CIDI MDD Diagnosis (*n* =577).

**MDD Diagnosis (Last 6 months)**				
**Depression level (PHQ-9)**	**CIDI negative**	**CIDI positive**	**Total**	**%**
Minimum (0–4)	385	1	386	66.9
Mild (5–9)	113	8	121	21.0
Moderate (10–14)	41	4	45	7.8
Moderate to severe (15–19)	15	8	23	4.0
Severe (20–23, 25–28)	2	0	2	0.3
Total	556	21	577	100.0

*MDD, Major depressive disorder; PHQ-9, Patient Health Questionnaire-9; CIDI, Composite international Diagnostic Interview*.

### Item Analysis and Reliability

Mean scores for all PHQ-9 items are depicted in Table 3. The two items reported most frequently were sleep problems and low energy. The least-recognized item was suicidal ideation, which also had the least corrected item-total correlation (0.35). Corrected item-total correlation was in the range of 0.35–0.59. As indicators of internal consistency, both McDonald's coefficient (ω), more suitable for scales with few response options, and Cronbach's coefficient (α), were calculated. Both indicators were acceptable: ω = 0.79 [95% CI: 0.75–0.80] and α = 0.78 [95% CI: 0.75–0.81], respectively. All items, if deleted, would decrease both coefficients for the total scale.

**Table 3:** PHQ-9 item level values and item-total correlations (*n* = 577).

				**If item dropped**	
**Questionnaire Item**	**Mean**	**SD**	**Item-total correlation**	**McDonald's ω**	**Cronbach's α**
1. Little interest or pleasure in doing things	0.37	0.72	0.52	0.77	0.75
2. Feeling down, depressed, or hopeless	0.55	0.85	0.59	0.76	0.74
3. Trouble falling or staying asleep, or sleeping too much	0.89	1.11	0.48	0.78	0.76
4. Feeling tired or having little energy	0.77	0.95	0.59	0.75	0.74
5. Poor appetite or overeating	0.52	0.90	0.42	0.78	0.77
6. Feeling bad about yourself or that you are a failure	0.28	0.67	0.51	0.77	0.76
7. Trouble concentrating on things	0.31	0.72	0.41	0.78	0.77
8. Moving or speaking so slowly that other people could have noticed	0.26	0.67	0.41	0.78	0.77
9. Thoughts that you would be better off dead or hurting yourself	0.06	0.31	0.35	0.79	0.78

### Structural Validity

**Figure 1:**
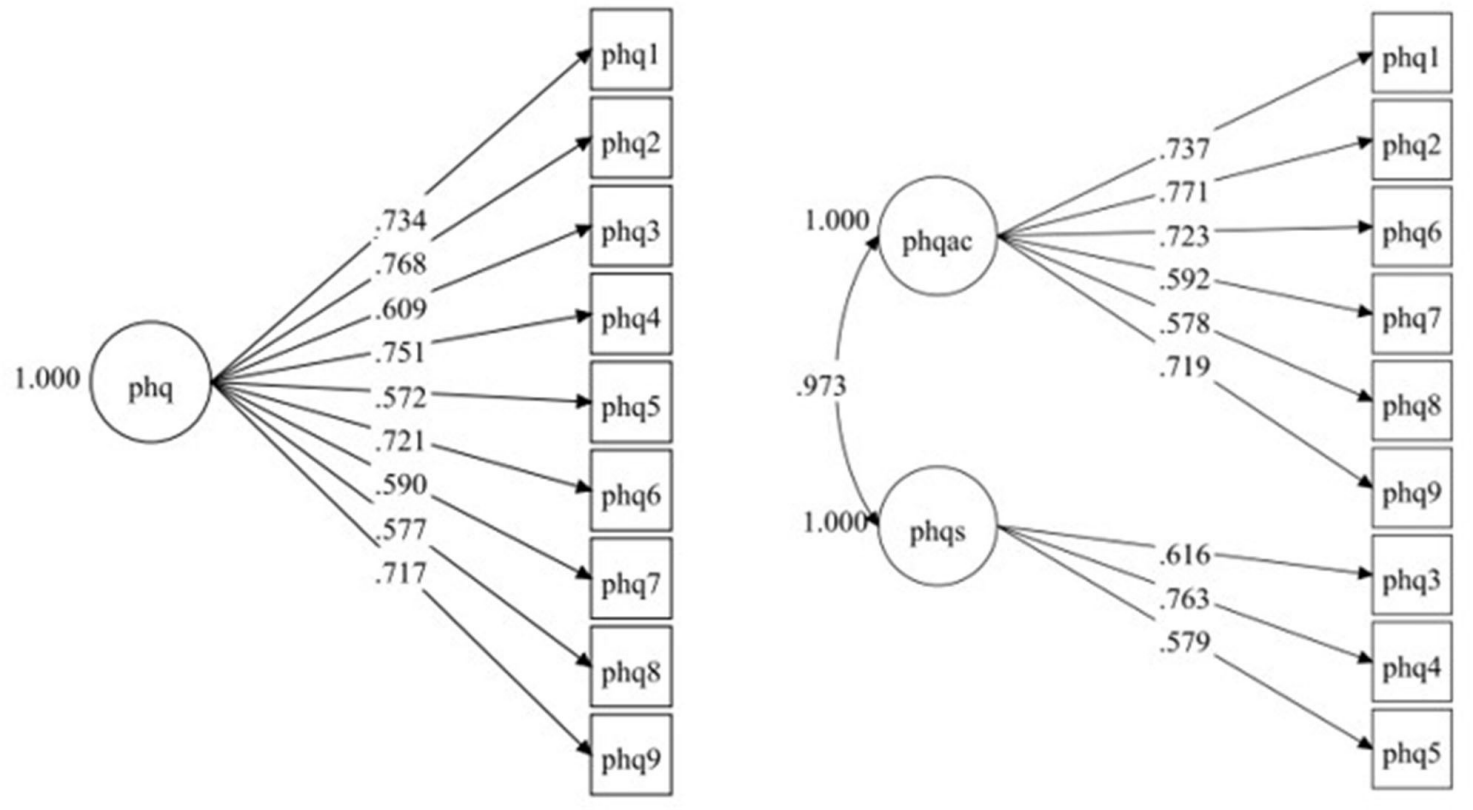
Factorial structure diagram for PHQ-9 scale. One & Two-factors solution. Standardized loadings.

One- and two-factor models of the PHQ-9 were assessed using CFA; the 1-factor solution indicated a good fit of the model to the data, (χ^2^= 51.91, *gl* = 27, *p* < 0.001; CFI = 0.99; TLI = 0.98; RMSEA = 0.04 [90% I.C. 0.02–0.06]); the 2-factor solution with three somatic items and six cognitive-affective items also indicated a good fit (χ^2^= 51.12, *gl* = 26, *p* < 0.001; CFI = 0.99; TLI = 0.98; RMSEA = 0.04 [90% I.C. 0.02–0.06]). In both models, although the chi-square value was significant, given the sample size, it is an expected result; CFI, TLI, and RMSEA values are within the recommended standards (i.e., CFI > 0.95, TLI > 0.95, RMSEA < 0.08) (47). A chi-square difference test was also performed to determine which of the two models has the best fit to the data. The nested comparison shows that the difference between both models was not significant (χ^2^ = 0.61, *gl* = 1, *p* = 0.43), suggesting that the more parsimonious 1 factor solution should be retained. Figure 1 shows the PHQ-9 factor structure for the 1-factor solution, where all the factor loadings were significant (*p* < 0.001) and >0.572. Figure 1 also reveals the PHQ-9 factor structure for the 2-factor solution, where all factor loadings were also significant (*p* < 0.001) and >0.578. The correlation between the somatic and the cognitive-affective latent factors was very high (*r* = 0.973, *p* < 0.001), suggesting that both factors would be overlapping.

### Criterion Validity

**Figure 2:**
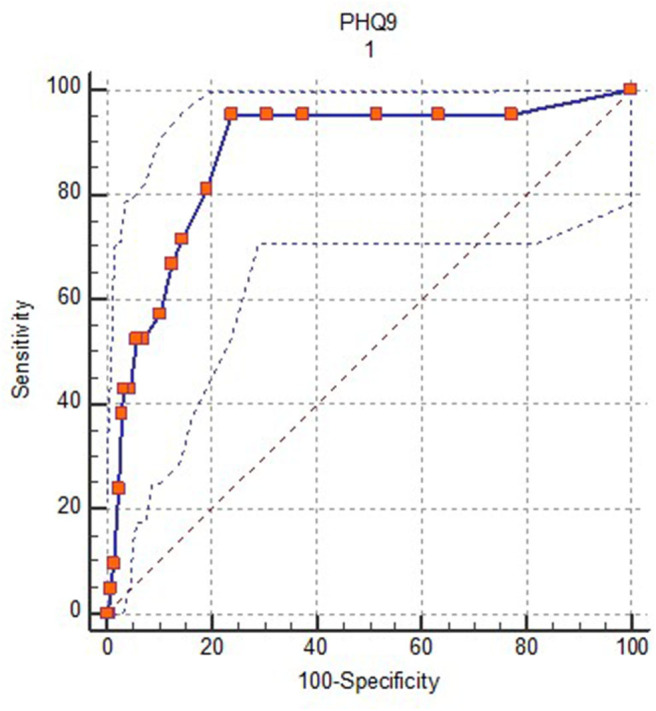
Receiver operation characteristic (ROC) curve for PHQ-9 vs. 6-month CIDI diagnosis of MDD.

According to the CIDI, 21 patients (3.6%) met the criteria for diagnosis of MDD in the last 6 months. Significant differences were observed in the average PHQ-9 scores between the participants with and without diagnosis (*M* = 11.19, *DT* = 4.89; *M* = 3.72, *DT* = 4.05, *t* (575) = 8.22, *p* < 0.001, *r* = 0.32). Figure 2 illustrates the ROC curve showing PHQ-9 performance in the identification of patients with MDD. The AUC was 0.88 (SE = 0.04, 95% CI: 0.85–0.90), which accounts for moderate accuracy (48).

Table 4 shows the sensitivity (Se), specificity (Sp), Youden J index, positive predictive value (PPV), negative predictive value (NPV), clinical utility index (CUI) and the likelihood ratio (LR) of different cut-off PHQ-9 scores in the diagnosis of MDD. The six-point cut-off score maximizes sensitivity and specificity values based on the Youden J index (sensitivity of 0.95 and specificity of 0.76). With this cut-off score, the PPV is 0.13 and the NPV is 0.99, and the positive and negative likelihood ratios are 3.95 and 0.06, respectively. Some authors (49–51) have pointed out limitations of the usual indicators used to estimate cut-off scores and have suggested alternatives such as the clinical utility index, which combines measures of occurrence and discrimination (52). This clinically relevant rule in accuracy is estimated by the clinical utility index positive (Se × PPV), and rule out accuracy is estimated through the clinical utility index negative (Sp × NPV). These measure the clinical value of a diagnostic test when applied in a specific setting, and can be graded using the following scale: < 0.5 poor, ≥ 0.5 < 0.64 fair, ≥ 0.64 < 0.81 good and ≥ 0.81 ≤ 1 excellent.

**Table 4:** Performance of PHQ-9 cut-off scores for detecting major depression.

**Cut-Off score**	**Sensitivity**	**Specificity**	**Youden J index**	**PPV**	**NPV**	**CUI+**	**CUI-**	**LR+**	**LR–**
≥ 6	0.95	0.76	0.71	0.13	0.99	0.12	0.75	3.95	0.06
≥ 7	0.81	0.81	0.62	0.14	0.99	0.11	0.80	4.25	0.24
≥ 8	0.71	0.85	0.56	0.16	0.99	0.11	0.84	4.90	0.33
≥ 9	0.67	0.87	0.54	0.17	0.99	0.11	0.86	5.3	0.38
≥ 10	0.57	0.90	0.47	0.17	0.98	0.10	0.88	5.48	0.48
≥ 11	0.52	0.93	0.45	0.22	0.98	0.11	0.91	7.47	0.51
≥ 12	0.52	0.94	0.46	0.26	0.98	0.14	0.92	9.10	0.51
≥ 13	0.43	0.95	0.38	0.26	0.98	0.11	0.93	9.16	0.6
≥ 14	0.43	0.97	0.40	0.32	0.98	0.14	0.95	12.54	0.59
≥ 15	0.38	0.97	0.35	0.32	0.98	0.12	0.95	12.46	0.64

*PPV, Positive predictive value; NPV, Negative predictive value; CUI+, Clinical utility index positive; CUI–, Clinical utility index negative; LR+, Likelihood ratio positive; LR–, Likelihood ratio negative*.

## Discussion

In this study, the psychometric properties of the PHQ-9 and their usefulness as a depressive disorder screening instrument are analyzed in a sample of older Chilean adults. Although this is not the only study of the functioning of the PHQ-9 that has been organized in Chile (23, 24), to our knowledge, it is the first study targeted at the older adult population, using a large sample of older adults who attend PHC centers. In addition, it has the advantage of having used the CIDI as a gold standard, which is an instrument widely applied in epidemiological studies due to its rigorous use of diagnostic criteria for established mental disorders.

The results show an adequate psychometric behavior of the PHQ-9 in the sample studied. Although the one dimension and two-dimensional solutions showed good a fit and high factor loadings, the scale appears to be one-dimensional given that the result of the chi-square difference test and the correlation between the non-somatic and somatic items was very high (*r* = 0.97), suggesting an overlap and supporting the notion of depression being a coherent unidimensional construct (11, 19). All items demonstrated loads >0.57, and corrected item-total correlations were >0.35. The internal consistency observed was also acceptable.

The PHQ-9 also reflected adequate sensibility and specificity values, comparable to those obtained in other studies (13, 16, 20). As is common with screening instruments used in contexts with low prevalence rates of the disorder (3.6% in this study), the best outcome of the PHQ-9 was in ruling out the presence of a depressive disorder rather than to positively confirm its presence. The PPV and NPV of scores between 6 and 11 are in the range of 0.13–0.22 and 0.99–0.98, respectively. These results suggest that in the population studied, a PHQ-9 high score should not be considered an indicator of probable a positive diagnosis but as an indication of the suitability of a new and more thorough evaluation; conversely, a low score confirms that the presence of an MDD is unlikely.

The cut-off score must be based on the objectives of those who use the instrument and the need to maximize detection or reduce the number of false positives (53, 54). Various indicators are considered for these purposes. One of the most used is the Youden index, which seeks to estimate the score that best combines sensitivity and specificity. In this study, the highest Youden J value is for the 6 cut-off score, which is in the lower range compared to other studies in elderly people (10, 13, 14, 17, 22). Nonetheless, some studies have presented similar results in elderly populations (16, 20, 21). It is not clear to us what to attribute this result to. Most of the studies mentioned above have been done with more specific samples of chronic patients than in this study, and with a higher prevalence of depressive disorder; these could be influencing factors. Cultural aspects may also play a relevant role, but it is unclear to which degree they affect this result. It should be considered that the Youden index is not necessarily the best criterion to select the cut-off point, since it does not consider the effect of prevalence and, therefore, the predictive value of the scores (52). Considering the clinical utility index, which combines measures of occurrence (Se/Sp) and discrimination (PPV/NPV), the best cut-off score should be higher. The results of this study suggest that the clinical utility index+ (adequate identification of cases) of the PHQ-9 in this simple is very low; in contrast, the clinical utility index- (discarding non-cases) is excellent, especially from 8 points onwards.

This study has some limitations. First, the performance of the PHQ-9 was limited by the low prevalence rate of MDD in the target population. However, it was slightly higher than that observed in the only Chilean epidemiological study of national scope, where the 12-month prevalence of MDD in the general population was 2.9% in those over 65 years of age, 1.6% among those of 65–75 and higher than 5.2% after 75 (4). The lower prevalence a of depressive disorder in older people, compared to that observed in other ages, has been confirmed in various studies (55). However, this does not diminish its clinical relevance, but, on the contrary, accentuates the challenge of its adequate detection and treatment. Second, the analysis in this study was done by contrasting two specific measures over time (PHQ-9 and CIDI) and assuming that the use of the CIDI as a gold standard and the DSM-IV diagnostic criteria in older adults results in the detection of “authentic” depressive disorders. This is questionable. The DSM-IV criteria may overestimate if they are used out of context (56, 57). On the other hand, the DSM-IV criteria (and those of the current DSM-V) do not facilitate the recognition of the specificities that depression can manifest in elderly people. There is some debate about the PHQ-9's applicability in persons suffering from multimorbidity, such as the elderly, where the somatic symptoms (fatigue, sleep disturbance, and poor appetite) may also be attributable to pain or another disease, and as a result may produce an overestimation of depression because of symptom overlap (58). The differential diagnosis between medical comorbidities and depressive symptoms was not considered in this study.

In summary, the results obtained indicate that the PHQ-9 is an instrument with adequate psychometric properties in elderly PHC users. In clinical settings, it should be considered that its greatest utility in this study population was to rule out the presence of depressive disorders rather than to identify possible cases. In people with scores above the cut-off point, it is essential to perform a new and more thorough evaluation.

## Data Availability Statement

The raw data supporting the conclusions of this article will be made available by the authors, without undue reservation.

## Ethics Statement

The studies involving human participants were reviewed and approved by Research Ethics Committee of Health Services of Concepción and Talcahuano. The patients/participants provided their written informed consent to participate in this study.

## Author Contributions

SS, JA, FC, CI, and PR prepared the study design. SS, FC, JA, and VB were involved in the selection of measurements. JA and CB prepared the data set, performed statistical analysis, and prepared the tables. CO polished and checked the manuscript. All authors have approved the final version of this manuscript.

## Conflict of Interest

The authors declare that the research was conducted in the absence of any commercial or financial relationships that could be construed as a potential conflict of interest.
